# Rebuilding northern foodsheds, sustainable food systems, community well-being, and food security

**DOI:** 10.3402/ijch.v72i0.21560

**Published:** 2013-08-05

**Authors:** S. Craig Gerlach, Philip A. Loring

**Affiliations:** 1 Center for Cross-Cultural Studies, University of Alaska Fairbanks, Fairbanks, AK, USA; 2 Alaska Center for Climate Assessment and Policy, University of Alaska Fairbanks, Fairbanks, AK, USA

**Keywords:** food security, Alaskan food systems, community health and well-being, food portfolios

## Abstract

**Background:**

Multiple climatic, environmental and socio-economic pressures have accumulated to the point where they interfere with the ability of remote rural Alaska Native communities to achieve food security with locally harvestable food resources. The harvest of wild foods has been the historical norm, but most Alaska Native villages are transitioning to a cash economy, with increasing reliance on industrially produced, store-bought foods, and with less reliable access to and reliance on wild, country foods. While commercially available market foods provide one measure of food security, the availability and quality of market foods are subject to the vagaries and vulnerabilities of the global food system; access is dependent on one's ability to pay, is limited to what is available on the shelves of small rural stores, and, store-bought foods do not fulfill the important roles that traditional country foods play in rural communities and cultures. Country food access is also constrained by rising prices of fuel and equipment, a federal and state regulatory framework that sometimes hinders rather than helps rural subsistence users who need to access traditional food resources, a regulatory framework that is often not responsive to changes in climate, weather and seasonality, and a shifting knowledge base in younger generations about how to effectively harvest, process and store wild foods.

**Objective:**

The general objective is to provide a framework for understanding the social, cultural, ecological and political dimensions of rural Alaska Native food security, and to provide information on the current trends in rural Alaska Native food systems.

**Design:**

This research is based on our long-term ethnographic, subsistence and food systems work in coastal and interior Alaska. This includes research about the land mammal harvest, the Yukon River and coastal fisheries, community and village gardens, small livestock production and red meat systems that are scaled appropriately to village size and capacity, and food-system intervention strategies designed to rebuild local and rural foodsheds and to restore individual and community health.

**Results:**

The contemporary cultural, economic and nutrition transition has severe consequences for the health of people and for the viability of rural communities, and in ways that are not well tracked by the conventional food security methodologies and frameworks. This article expands the discussion of food security and is premised on a holistic model that integrates the social, cultural, ecological, psychological and biomedical aspects of individual and community health.

**Conclusion:**

We propose a new direction for food-system design that prioritizes the management of place-based food portfolios above the more conventional management of individual resources, one with a commitment to as much local and regional food production and/or harvest for local and regional consumption as is possible, and to community self-reliance and health for rural Alaska Natives.

Food security is most commonly defined as whether or not people have equitable physical and economic access to sufficient and safe foods (1). In the context that we use it here, however, food security means more than simply whether or not sufficient food is being produced or harvested in a “one-size-fits-all” food-to-nutrition relationship (2), and expands to include all of the various ways in which a food system supports health in the biophysical, social and ecological dimensions (3, 4). These include the importance of culturally preferred foods, food choice, local perceptions of hunger, uncertainty and worry about food safety or food shortages, and any other psychosocial, social, cultural or environmental stresses that result from the process of putting food on the table. In rural, predominately Alaska Native communities, for example, wild fish and game, “country foods,” are important for food security, not just because they are readily available and of historical significance, but also because they are important to the preservation and transmission of traditions and cultural practices, for the maintenance of social networks and interpersonal relationships, and for supporting individual and community sense of self-worth and identity (3, 5).

Yet, food insecurity in Alaska and the Canadian North is a growing problem (3, 6, 7). According to the United States Department of Agriculture (USDA), Alaska currently has a food insecurity rate of 14.5%, lower than the nationwide average of 16% (8), although rates may be much higher for many rural communities. The non-profit group Feeding America estimates that some rural parts of the state currently experience food insecurity rates as high as 30%, with children among those most directly affected (9). One challenge in measuring food security in the North, however, is that the standardized, validated research protocols such as those used by the USDA are not necessarily appropriate for remote communities. For example, the USDA Food Insecurity protocol focuses on the availability of money to buy food, but in Alaska where subsistence foods play an important role for households in both rural and urban settings, the USDA tool does not capture this aspect well, if at all (10, 11). Similarly, the USDA protocol also invokes the concept of a “balanced” diet, but this is confusing to many in Alaska where traditional foodways are fluid, flexible and highly seasonal in nature. Use of the word “balanced” might also lead some respondents to self-assess against their perceptions of government standards for nutrition, rather than in terms of their own traditions, preferences and conceptions of health.

## Cultural and nutritional transitions

Our research has investigated the multiple drivers and determinants of food security and insecurity in the North, and finds that, while circumstances and challenges vary from place to place, foodshed to foodshed, some general themes emerge as they connect food, livelihoods, individual and community health. Regardless of the metric chosen, indigenous peoples across the North American Arctic are “coming out of their traditional foodsheds,” with the use of country foods declining, and being replaced instead with market foods that, while readily available, are both expensive and generally poor in nutritional quality by comparison (12–14). Consistent with this transition, people are increasingly experiencing a host of diet-related and community-based health problems, including but not limited to higher incidences of colorectal cancer, obesity and diabetes (15, 16), as well as to various chronic psychological and psychosocial problems, such as domestic violence, alcoholism, depression and drug abuse (17). While direct causality among one or more of these dietary changes and health trends are difficult to clinically establish, the consensus among many health researchers, practitioners and local people is that solutions for these problems are best situated in local food-system reform and revitalization (18, 19).

Part of the challenge with respect to enhancing food security in Alaska, which we draw on as an example that no doubt has parallels to communities in Arctic Canada as well, relates to the limited capacity of the contemporary northern food production and distribution system. Despite active local food movements in many parts of Alaska (20), only an estimated 2–5% of agricultural products consumed in Alaska are actually produced in Alaska. Agricultural production is limited by various factors, not least of which is a paucity of farms, farmers, and in-state infrastructure for food processing and distribution (21, 22). Similarly, while the commercial seafood industry is robust and thriving, providing 50% of US wild landings (23), very little of this commercial catch is marketed in Alaska, and is instead fed into national and global seafood and commodity markets. Specifics are rare regarding the quantity and origin of seafood that is actually consumed directly by Alaska Natives (21), but even in the iconic fishing communities featured in this research, most grocers do not offer a fresh seafood counter. Recently, the noticeable disparities in who benefits from Alaska's commercial fisheries have led some to question the social justice implications of their widespread reputation of sustainability (24).

## Discussion: a portfolio approach to rebuilding northern foodsheds

In Alaska and elsewhere, there is no shortage of good ideas for how Northern people can enhance local and regional food security by rebuilding food systems around such values as food sovereignty and self-reliance (25). The portfolio approach to food-system design and management is a new direction that we are now exploring in on-going research as we believe it to be relevant to the revitalization and long-term sustainability of local and regional food systems (26). Village gardens are being successfully restored to the food-system portfolio for many Alaska Native villages, especially up and down the Yukon and Tanana Rivers, and these complement rather than replace subsistence, and in so doing, diversify the food-system options and improve the food security situation.

The intent of managing for a portfolio of food resources together is to foster a system with the built-in flexibility needed so that people can respond to variability and change in the availability of specific food resources, whether this reflects a year's salmon return or the return of a potato or garden crop, and in ways that enhance rather than diminish local and regional food security. In other words, decisions regarding the production and marketing of food resources need to be made in a flexible, effective and regionally tailored fashion, much in the way that many indigenous societies adapt culturally to environmental variability and change through flexible subsistence calendars that incorporate multiple primary and secondary food options (27, 28).

## Conclusion

Residents of rural Alaska who continue to engage in subsistence activities still do, to some extent, maintain a portfolio approach to food security, though their flexibility today is constrained by contemporary management approaches that focus on single-species outcomes, and by a patchwork of land tenure that severely restricts hunter and fisher flexibility when responding to change (26, 29). More integrated and holistic approaches to managing wild fish and game resources that take a food-system approach should be explored. However, we also note that the portfolio approach is just one step, one that must be accompanied by a commitment to the social justice as well as to the food production and harvest aspects of the food system, and one that ensures that Alaskan Native are fed before food resources are marketed elsewhere. Otherwise, we argue, the sustainability and health of both Alaska's local communities and their highly valued renewable food resources will remain uncertain.
